# The Effect of Lithocholic Acid on the Gut-Liver Axis

**DOI:** 10.3389/fphar.2022.910493

**Published:** 2022-07-07

**Authors:** Wei Sheng, Guang Ji, Li Zhang

**Affiliations:** Institute of Digestive Diseases, Longhua Hospital, Shanghai University of Traditional Chinese Medicine, Shanghai, China

**Keywords:** lithocholic acid, natural products, intestinal barrier, gut microbiota, bile acid receptors

## Abstract

Lithocholic acid (LCA) is a monohydroxy bile acid produced by intestinal flora, which has been found to be associated with a variety of hepatic and intestinal diseases. LCA is previously considered to be toxic, however, recent studies revealed that LCA and its derivatives may exert anti-inflammatory and anti-tumor effects under certain conditions. LCA goes through enterohepatic circulation along with other bile acids, here, we mainly discuss the effects of LCA on the gut-liver axis, including the regulation of gut microbiota, intestinal barrier, and relevant nuclear receptors (VDR, PXR) and G protein-coupled receptor five in related diseases. In addition, we also find that some natural ingredients are involved in regulating the detoxification and excretion of LCA, and the interaction with LCA also mediates its own biological activity.

## Introduction

Bile acids (BAs) have been shown to play an irreplaceable role in many diseases (Fuchs and Trauner, 2022). The size and composition of the BA pool is the target for the treatment of a series of hepatoenteric diseases including inflammatory bowel disease (IBD), primary biliary cholangitis (PBC) and non-alcoholic steatohepatitis (NASH) (Ridlon et al., 2006; Kim et al., 2019). Lithocholic acid (LCA), also known as 3α-hydroxy-5β-cholan-24-oic acid, is a monohydroxy BA produced from chenodeoxycholic acid (CDCA) or ursodeoxycholic acid (UDCA) by the action of intestinal bacteria (Fiorucci et al., 2018; Marion et al., 2019). LCA acts as a detergent to solubilize fat for absorption in intestine, but is considered to be toxic for hepatocytes (Hofmann, 2004; Doden et al., 2021). Particularly, LCA has also been considered to be a carcinogen, high concentrations of LCA induce oxidative stress and DNA damage, and promote tumor development by inhibiting the action of DNA repair enzymes and promoting the proliferation of cells (Bernstein et al., 2005; Ridlon et al., 2016b; Nguyen et al., 2021). Contrary to the previous reports, recent studies revealed that LCA might be a protector in restraining hepatic and intestinal inflammation (Hamilton et al., 2007; Ward et al., 2017; Sinha et al., 2020). Some other studies have found that LCA also potentiates anti-aging and anti-tumor effects (Burstein et al., 2012; Arlia-Ciommo et al., 2014; Arlia-Ciommo et al., 2018). For example, galactosylated poly (ethyleneglycol)-LCA, a nanoparticle formulation can selectively induce apoptosis in hepatocellular carcinoma cells without adverse effects on normal liver cells (Gankhuyag et al., 2015).

Many natural extracts from plants or animals can regulate intestinal microbiota, BA metabolism and related signaling pathways (Han et al., 2017; Hu et al., 2021). Recent studies have shown that natural components are involved in the regulation of LCA production, detoxification and related signaling pathways. For example, grape seed proanthocyanidin and polyphenol extracts from pomegranate are reported to promote the production of LCA (Yang et al., 2018; Wu et al., 2021). Glycyrrhizin and oleanolic are found to accelerate LCA detoxification by up-regulating the expression of detoxification enzymes (Wang et al., 2012; Chai et al., 2015). Kaki-tannin increases the fecal excretion of LCA (Nishida et al., 2021). Bufalin enhances the activation of vitamin D receptor (VDR) via LCA binding (Nakano et al., 2005). Furthermore, natural ingredients modified by LCA can enhance their albumin affinity and stability, thus continuously exhibiting effective concentrations and better therapeutic effects (Han et al., 2018; Sreekanth et al., 2021).

Collectively, LCA undergoes enterohepatic circulation along with other BA species, and affects the gut-liver axis and the development of related diseases. A series of natural ingredients are involved in the regulation of LCA metabolism. Therefore, this review will discuss the new understanding and update the progression of the functions of LCA, and how natural ingredients modulate LCA metabolism.

## The Synthesis of LCA

BAs are synthesized from cholesterol in the liver via both the classic pathway and the alternative pathway. Currently, 14 essential enzymes are identified in converting cholesterol to primary BAs in the liver (Ridlon et al., 2006). In the classic pathway, the rate-limiting enzyme cholesterol 7α-hydroxylase (CYP7A1) is mainly responsible for the cholic acid (CA) and CDCA synthesis. As far as the alternative pathway, the main rate-limiting enzyme is sterol 27-hydrolase (CYP27A1), which responds to CDCA production (Fuchs and Trauner, 2022). It is reported that extracts from some natural products can promote LCA production via activating intestinal farnesoid X receptor (FXR) signaling and increasing the gene expression of BA alternative pathway synthetases CYP7B1 and CYP27A1 (Wu et al., 2021). Polyphenol extracts from pomegranates also increase the expression of CYP7A1 and CYP7B1, and increase the expression levels of some hepatic genes involved in fatty acid β -oxidation (Yang et al., 2018).

**FIGURE 1:**
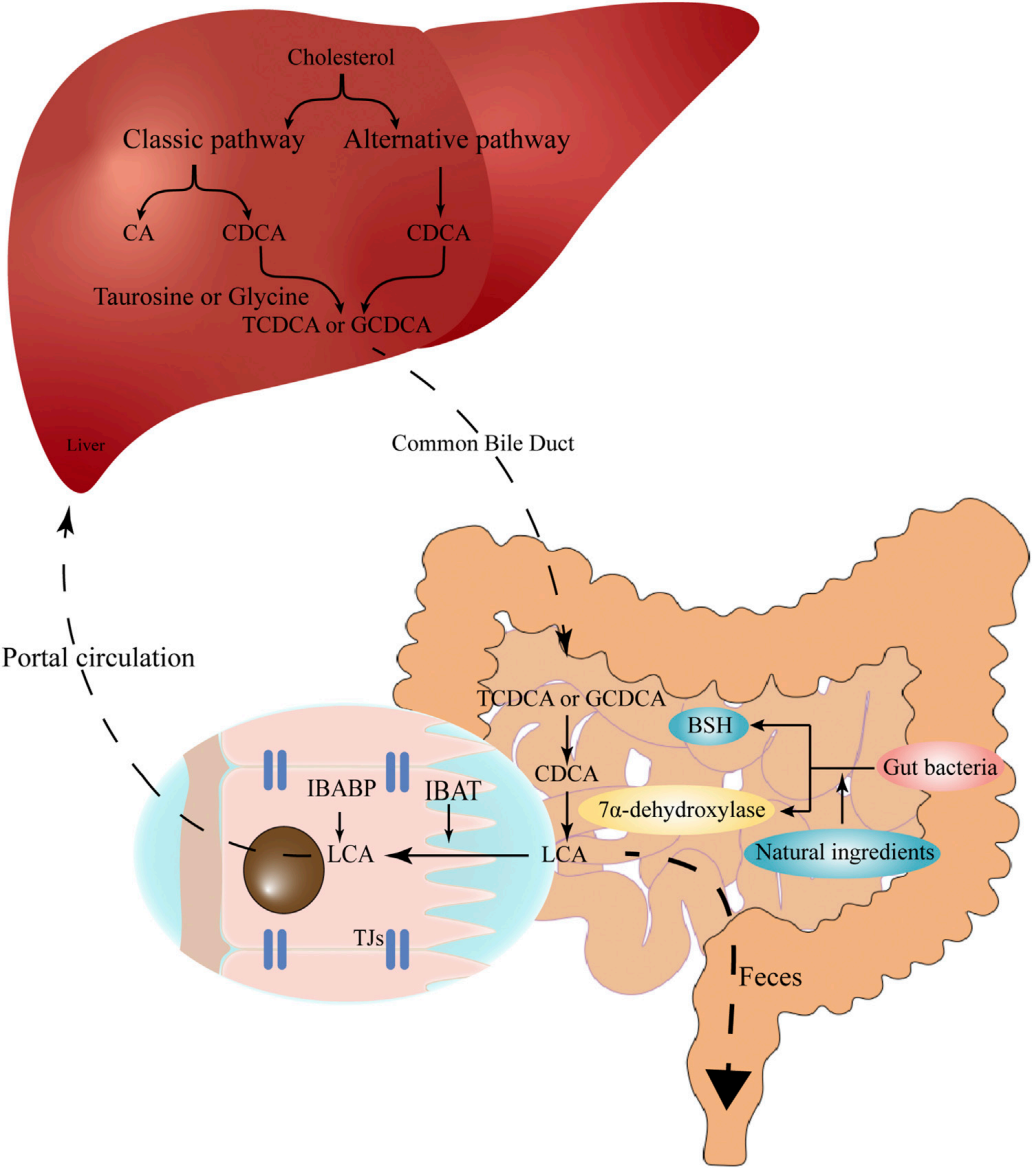
The synthesis and enterohepatic circulation of LCA. Cholesterol is converted into primary BAs (CA and CDCA) under the action of CYP7A1, CYP27A1, CYP8B1 and related enzymes in hepatocytes. Primary BAs conjugate with taurine or glycine, temporarily store in the gallbladder, and then are excreted into the gut upon food intake. In the gut, the conjugated BAs are subsequently uncoupled by gut bacteria and converted to secondary BAs (e.g., LCA) via 7α-dehydroxylation. The high-affinity IBAT actively transports conjugated and unconjugated LCA from the intestinal lumen to the ileocytes, where they are bound to IBABP and eventually exported across the basement membrane to the portal circulation and back to the liver. Natural ingredients can up-regulate the expression of bile salt hydrolase and 7α-dehydroxylation, therefore promote the production of LCA. LCA, lithocholic acid; BAs, bile acids; CA, cholic acid; CDCA, chenodeoxycholic acid; CYP7A1, cholesterol 7α-hydroxylase; CYP27A1, sterol 27-hydrolase; CYP8B1, 24-hydroxycholesterol-7α-hydroxylase; IBAT, intestinal bile acid transporter; IBABP, ileal bile acid binding protein.

These primary BAs (CA and CDCA) are then conjugated with taurine or glycine to form conjugated BAs in the liver, the conjugation can reduce the pK_a_ and increase the solubility of BAs (Ridlon et al., 2006). Conjugated primary BAs are then temporarily stored in the gallbladder and transported into the gut upon food intake for emulsifying dietary lipids and fat-soluble vitamins (Doden et al., 2021). In the gut, the conjugated primary BAs are subsequently uncoupled by intestinal bacteria via the action of bile salt hydrolase (BSH) (Ridlon et al., 2016a), and then converted to secondary BAs via 7α-dehydroxylation (Dawson and Karpen, 2015). The production of LCA is mainly attributed to 7α-dehydroxylase-producing bacteria such as *Clostridium* and *Eubacterium* in human, while in rodents, LCA can also be produced by UDCA through 7β-dehydroxylase (Fiorucci et al., 2018). Furthermore, a Chinese medicinal herb, vinegar frankincense is reported to activate the BSH activity and LCA production, thus promote the conjugation of BAs in the liver (Peng et al., 2022) (Figure 1). Therefore, the cellular cholesterol, primary BA excretion, as well as the structure of gut microbiota conjointly determine the production of LCA in the gut.

## The Enterohepatic Circulation and Excretion of LCA

The size of the BA pool, under physiological conditions, is defined as the total amount of BAs in the enterohepatic circulation (Sarathy et al., 2017). Secondary BAs such as deoxycholic acid (DCA), LCA and their derivatives are important components of the circulating BA pool (Arab et al., 2017), accounting for more than 90% of the BA pool in the intestine and more than 25% in the gallbladder (Ridlon et al., 2006). The total BA pool is about 1,300–3,650 mg, while the physiological concentration of LCA is about 50–150 mg (Sarathy et al., 2017). In the enterohepatic circulation, LCA is mainly detected in the portal vein as conjugates, such as glyco-LCA (GLCA) and tauro-LCA (TLCA) (Bansal and Lau, 2019), the free form of LCA can reach 0.5 μM in the peripheral circulation (Steiner et al., 2011). During the enterohepatic circulation, the high-affinity intestinal bile acid transporter (IBAT) actively transports conjugated and unconjugated BAs from the intestinal lumen to the ileocytes, where they are bound to ileal bile acid-binding protein (IBABP), exported across the basement membrane to the portal circulation, and eventually back to the liver (Dawson and Karpen, 2015; Ridlon et al., 2016b). When LCA reenters the liver via the enterohepatic circulation, it is mainly converted to taurine and glycosylated conjugates, and conjugated LCA is then converted to 3-sulfate and 3-glucosylate LCA via sulfotransferase (SULT) and UDP-glucuronosyltransferase (Stiehl, 1974; Dionne et al., 1994).

About 2 g of the BAs undergo continuous enterohepatic circulation 8–10 times per day, while 95% BAs can be re-absorbed in each cycle, about 600–800 mg of BAs directly enter the large intestine and are excreted via the feces (Fiorucci et al., 2018; Doden et al., 2021). The fecal-dependent excretion is mainly attributed to the conjugation of LCA. Although the free form of LCA is insoluble in water and effectively precipitated in the acidic environment of the colon, the conjugated LCAs are soluble in acidic pH solutions and are resistant to precipitation (Hofmann and Roda, 1984). In addition, binding to VDR leads to enzymatic sulfidation of LCA, which increases LCA hydrophilicity and inhibits the passive absorption by the colonic epithelium (Huang et al., 2010; Ridlon et al., 2016b). Supplementation of natural products may also affect the excretion of LCA. Kaki-tannin (an extract from persimmon) preferentially binds to monohydroxy LCA and promotes its excretion (Nishida et al., 2021). Ethanolic extract of Fructus schisandrae enhances the excretion of BAs to the intestine and feces and restores the structure of intestinal flora that is destroyed by excess LCA (Li et al., 2020).

## Detoxification of LCA

The liver is the primary place for drug metabolism and elimination, including processes such as drug uptake, phase I and II reactions, and excretion (Ruiz et al., 2013). The biotransformation can effectively reduce the toxicity of various endogenous and exogenous toxins. After reabsorption to the liver, LCA initially undergoes a phase I metabolic reaction catalyzed by cytochrome P450 (CYP450) enzymes in hepatocytes and a subsequent phase II reaction in which the 3α-hydroxyl site is conjugated to sulfate (Kitada et al., 2003; Hartley et al., 2004). CYP450-mediated hydroxylation is considered to be an effective detoxification mechanism in rodents and monkeys, whereas human LCA is detoxified mainly through sulfated conjugation (Hartley et al., 2004). In addition, studies on Caco-2 cells (human colon cancer cell line) showed that LCA is rapidly metabolized to the less toxic metabolites by sulfation during its first pass through the human liver, whereas other BA species lack such an efficient sulfation ability (Gadacz et al., 1976; Hofmann, 2004).

However, recent studies have shown that SULT2A1-catalyzed LCA sulfation is also a predominant detoxification pathway in mouse liver, whereas CYP3A is only involved in the formation of 6α- and 6β-hydroxylation of LCA microsomes as an alternative pathway (Kitada et al., 2003). In Caco-2 cells, LCA is mainly detoxified in the sulfonation reaction catalyzed by SULT2A1 to form LCA-S (Huang et al., 2010; Kurogi et al., 2011), while GLCA and TLCA undergoes sulfonation to form glycolithocholic acid sulfate (GLCA-S) and taurolithocholic acid sulfate (TLCA-S), respectively (Chen and Segel, 1985). In rat liver, LCA is mainly transformed into murideoxycholic acid (MDCA) under the action of 6β-hydroxylation of CYP2C, CYP3A and CYP2D1 (Zimniak et al., 1989; Dionne et al., 1994; Deo and Bandiera, 2008). In addition, the most predominant subfamily of CYP450 enzymes is CYP2C in rat liver, whereas in the human liver, CYP3A is the predominant enzyme for the formation of LCA metabolites, such as LCA 6α- and 6β-hydroxylation (Araya and Wikvall, 1999; Xie et al., 2001; Deo and Bandiera, 2008). LCA can be sulfated in Caco-2 cells, this sulfation appeared to be an important mechanism for blocking LCA-induced malignant epithelial phenotype, as LCA alone induces a tumor-invasive phenotype in Caco-2 cell, whereas its sulfate counterpart LCA-S does not (Halvorsen et al., 2000). Once the sulfation is impaired, excessive accumulation of LCA may lead to associated adverse effects (e.g., cholestasis) (Bansal and Lau, 2019).

Many natural components can participate in the process of LCA detoxification and reduce LCA-induced liver damage. For example, glycyrrhizin, the main component of licorice, can promote the detoxification process of LCA by accelerating CYP3A catalyzation (Wang et al., 2012). Both juniperus procera extract and artemisinin treatment prevent hepatotoxicity and cholestasis caused by LCA exposure (Alkhedaide, 2018; Alkhedaide et al., 2018). Lysimachia christinae Hance also improves LCA-induced cholecystitis (Yang et al., 2011). Oleanolic acid isolated from swede has also been shown to promote the expression of detoxification enzymes CYP3A, UGT2b, and SULT2A1, and significantly reduce the serum BA levels (Chai et al., 2015).

## LCA and the Intestinal Environment

### LCA and Intestinal Flora

BAs are steroid molecules that modulate the host and the microorganisms that they carry, and their antimicrobial properties inhibit the overgrowth of intestinal bacteria (Zhou and Hylemon, 2014). Similarly, as the second “genome”, the microbiome content and their genetic structure also influence the composition of the BA pool (Begley et al., 2005; Ridlon et al., 2016b). BAs undergo different biotransformation, in the liver, predominant BAs are conjugative and oxidative, whereas they are prone to be hydrolytic and reductive in the gut by the action of microbiota (Ridlon et al., 2016b). Secondary BAs are the most concentrated bacterial-derived intestinal metabolites (Sinha et al., 2020). Clostridium and Eubacterium expressing 7α-dehydroxylase is known to promote LCA synthesis (do Nascimento et al., 2015). The enrichment of 7a-dehydroxylase producing-bacteria might be a key rate-limiting enzyme in the formation of LCA (Wells et al., 2000). The abundance of *Clostridium* and *Eubacterium* in the colon is relatively low, whereas the amount of primary BAs to be treated is large (Ridlon et al., 2006). Many natural ingredients have been reported to up-regulate the expressing 7α -dehydroxyase producing gut microbiota and promote LCA production. For example, capsaicin significantly increases the abundance of *Bacteroides* genera, which is associated with LCA production (Hui et al., 2020a). Curcumin increases the relative abundance of *Lactobacillus*, the prominent BSH-producing bacteria (Han et al., 2021). In mice with dextran sulfate sodium-induced colitis, dihydromyricetin also significantly increases the ratio of *Lactobacillus* and *Akkermansia* genera, thus increasing the intestinal LCA species (Dong et al., 2021).

BAs can cause non-specific damage to the ***cytoplasmic*** membrane of bacteria due to their ***lipophilicity*** and decontamination ability (Gonzalez et al., 2020). It should be noted that among these amphiphilic molecules, LCA is the most lipophilic and hydrophobic BA (Goldberg et al., 2010; Ward et al., 2017). Moreover, LCA also exhibits specific antibacterial effects against certain bacteria. LCA at 32 mg/L rapidly destroyed *Helicobacter pylori* and prevented its growth, more importantly, LCA even shows synergistic effects with clarithromycin or levofloxacin (Gonzalez et al., 2020). Furthermore, in the evaluation of the function of LCA and its metabolites, Nascimento *et al.* found that these metabolites not only show significant antibacterial activity against *Escherichia coli*, *Staphylococcus aureus*, *Bacillus cereus*, and *Pseudomonas aeruginosa*, certain LCA metabolites could even synergistically enhance the function of antibiotics (do Nascimento et al., 2015).

The enrichment of bacteria that promote secondary BA production is reported to be reduced in patients with ulcerative colitis, while secondary BAs supplementation could alleviate intestinal inflammation (Sinha et al., 2020). In addition, dihydromyricetin supplementation also restores the relevant dysbiosis and increases the gastrointestinal levels of CDCA and LCA, thereby improving the metabolism of BAs and alleviating the colitis in mice (Dong et al., 2021). Furthermore, compared to healthy children, the abundance of BSH and 7α-dehydroxylase-producing bacteria *Eubacterium* and *Ruminococcus* in children with NAFLD is significantly decreased, which is in correlation with the concentration of fecal LCA (Yu et al., 2021). In addition, *Ruminococcus* is also significantly reduced in patients with irritable bowel syndrome with diarrhea (IBS-D), which may cause an increase in primary BAs (CA and CDCA) and a decrease in secondary BAs (LCA and UDCA) in feces (Wei et al., 2020). The clinical significance of these findings lies in the fact that we can reduce the intestinal inflammatory response in IBD by specifically restoring the original levels of secondary BAs, either through direct BA supplementation or indirectly bacteria modulation (Sinha et al., 2020).

Diet is another critical modulator of gut microbiota and BAs (Ridlon et al., 2014). A hydrolysable protein diet is found to diminish chronic enteropathy (e.g., Crohn’s disease), possibly through inhibiting the growth of pathogenic bacteria such as *Escherichia coli* and *Clostridium perfringens*, and promoting secondary BA production (Wang et al., 2019). High-fat diet or high-animal protein diet may stimulate BA excretion into the intestine, increase BSH-producing bacteria, and lead to LCA accumulation (David et al., 2014; O'Keefe et al., 2015; Ridlon et al., 2016b). Consuming excessive animal protein or fat potentiate risks for CRC, which may be associated with a high concentration of LCA in the gut (Durko and Malecka-Panas, 2014). Another study pointed out that a high-fat and high-sugar diet-induced dysbiosis subsequently initiates BA alteration and liver injury in mice (Iwamoto et al., 2021). Therefore, there is a dynamic balance between the diet, gut microbiome and the size or composition of the BA pool.

### LCA and Intestinal Barrier

The intestinal barrier is mainly composed of a biological barrier, mechanical barrier, immune barrier and chemical barrier. Among them, the mechanical barrier is the most important line of defense in the intestinal epithelial barrier. The mechanical barrier is composed of mucosal epithelial cells, lateral tight junction proteins (TJs) and a basement membrane, which can protect against microorganisms, pathogens and potentially toxic substances. Intestinal mucosal barrier dysfunction can lead to increased permeability and inflammatory-related diseases, including IBD, necrotizing enterocolitis, and NASH (Wang et al., 2015; Holmberg et al., 2018). TJs, also known as interepithelial cell junctions, are located in the most apical region of cell-cell contacts, and are composed of a group of transmembrane proteins (e.g., Occludin and Claudin-1), as well as peripheral membrane proteins (e.g., Zonula occludens-1, E-cadherin) (Sanchez de Medina et al., 2014; Chen et al., 2015; Otani and Furuse, 2020). Studies have shown that altered expression and localization of TJs are associated with impairment of intestinal epithelial barrier induced by proinflammatory cytokines (PiC) (Suzuki et al., 2011).

Under physiological conditions, conjugated BAs are completely ionized and impermeable to epithelial cell membranes such as hepatocyte membranes and bile duct cell membranes (Ridlon et al., 2016b). Once the intestinal barrier is damaged, various toxic substances (e.g., endotoxins, BAs, bacterial debris) can enter the circulation, thus causing intestinal and systematic inflammation. Overwhelm BAs are usually assumed to be toxic to the physiological intestinal structure. Surprisingly, CDCA and its 7α-deoxy derivative LCA may have exactly the opposite effect on the epithelial integrity of human colonic cells (Sarathy et al., 2017). CDCA stimulates the secretion of Cl^−^ (Dharmsathaphorn et al., 1989; Ao et al., 2013), whereas physiological concentration (0.005–0.05 mmol/L) of LCA inhibits Cl^−^ secretion in the colon (Ao et al., 2016). This finding suggests that a low dose of LCA has potential in the treatment of diarrhoeal diseases. Conversely, high concentrations of LCA may contribute to the development of constipation. This is also evidenced by the improvement in constipation observed in rats with constipation treated with rhubarb, because rhubarb treatment reduced intestinal LCA levels (Yang et al., 2022).

In TNF-α stressed Caco-2 cells, LCA partially reversed the decrease in transepithelial electrical resistance (TEER) and the increase in FITC-Dextran flux, and increased the expression of TJs (Yao et al., 2019). LCA alone had no effect either on TEER or paracellular permeability, however, LCA significantly attenuated (≥80%) the effect of PiC on intestinal barrier permeability (Sarathy et al., 2017). Further research has shown that LCA also attenuated intestinal barrier permeability induced by CDCA combined with PiC (Sarathy et al., 2017). LCA significantly improves TEER of co-cultured Caco-2 and HT29-MTX-E12 cells, and increases the expression of TJs (van der Lugt et al., 2022). Our recent work revealed that Traditional Chinese medicine prescription Jiangzhi Granule attenuates NASH through secondary BAs (e.g., LCA and keto-LCA) mediated VDR activation (Cao et al., 2022). Paradoxically, another Caco-2 cell assay showed that a higher concentration of LCA (100–200 μM) down-regulates the expression of genes encoding TJs and up-regulates epidermal growth factor receptor (EGFR) as well as Src protein. Inhibition of EGFR and Src expression eliminates LCA-induced Zonula occludens-1 downregulation, suggesting that LCA impairs intestinal barrier function by enhancing EGFR-SRC pathway (Pi et al., 2020). However, a recent study showed that piperine can inhibit EGFR and Src and block LCA-stimulated IL-8 expression (Li et al., 2022). Furthermore, direct exposure to secondary BAs rather than sulfating secondary BAs resulted in reduced PiC production, suggesting that sulfation of secondary BAs might dimmish their anti-inflammatory effects (van der Lugt et al., 2022). Therefore, under certain conditions, LCA may play a protective role in maintaining the stability of intestinal TJs and intestinal barrier permeability. Studies related to LCA and intestinal barrier are summarized in Table 1.

**TABLE 1:** Effects of LCA on intestinal barrier in different models.

Models	Effects of LCA on Intestinal Barrier	Refs
Human colonic T84 cells	Inhibition of Cl^−^ secretion	Ao et al. (2016)
TNF-α-stressed Caco-2 cells	Alleviation of the decrease in TEER and the increase in FITC-Dextran flux	Yao et al. (2019)
	Expression of TJs increase	
Human colonic T84 cells	No effect either on TEER or paracellular permeability	Sarathy et al. (2017)
	Attenuation the effect of PiC on intestinal barrier permeability	
Co-cultured Caco-2 and HT29-MTX-E12 cells	Improvement of TEER	van der Lugt et al. (2022)
	Expression of TJs increase	
Caco-2 cells	VDR activation and expression of TJs increase	Cao et al. (2022)
Caco-2 cells	Down-regulation of TJs expression	Pi et al. (2020)
	Up-regulation of EGFR and Src protein	

TEER, transepithelial electrical resistance; EGFR, epidermal growth factor receptor.

## Receptors for LCA

LCA mediates its functions through corresponding receptors, including VDR, G protein-coupled receptor 5 (TGR5), FXR and pregnane X receptor (PXR) (Lajczak-McGinley et al., 2020).

### LCA and VDR

VDR is a member of the nuclear receptor superfamily that primarily mediates the biological activity of 1α,25-dihydroxyvitamin D3 (1,25(OH)_2_D_3_), and plays an important role in the regulation of calcium homeostasis, cell growth, immune response and cardiovascular functions (Nishida et al., 2020). In 2008, it is firstly reported that LCA could conduct some of the functions that are associated with vitamin D in a vitamin D deficiency rat model (Matsubara et al., 2008). Initially, LCA was found to be a selective VDR agonist (Makishima et al., 2002). However, in contrast to 1,25(OH)_2_D_3_, which acts mainly in the upper intestine, LCA acts mainly in the lower intestine, especially the ileum (Makishima et al., 2002; Ishizawa et al., 2018). VDR shows a high affinity for LCA in most animal species, but the ability of LCA to act as a VDR ligand appears to be present only in higher vertebrates such as birds and mammals (Kollitz et al., 2016). Interestingly, studies have found that bufalin (an active ingredient in toad venom) does not directly activate VDR, but regulates LCA and enhances LCA-mediated VDR activation (Nakano et al., 2005).

The binding of LCA to the VDR activates a signaling cascade that leads to VDR-dependent sulfation, and the sensitivity of the LCA and its metabolites to VDR is at least one magnitude higher than that of other nuclear receptors (Makishima et al., 2002). The sulfation that occurs in the intestinal epithelium also contributes to the detoxification of LCA. Intestinal VDR deficiency increased LCA-induced hepatic necrosis and inflammation (Cheng et al., 2014). Notably, Makishima (Makishima et al., 2002) and Cheng (Cheng et al., 2014), further demonstrated that activation of VDR by LCA or vitamin D subsequently increases the expression of CYP3A, which detoxifies LCA in the liver and intestine.

**FIGURE 2:**
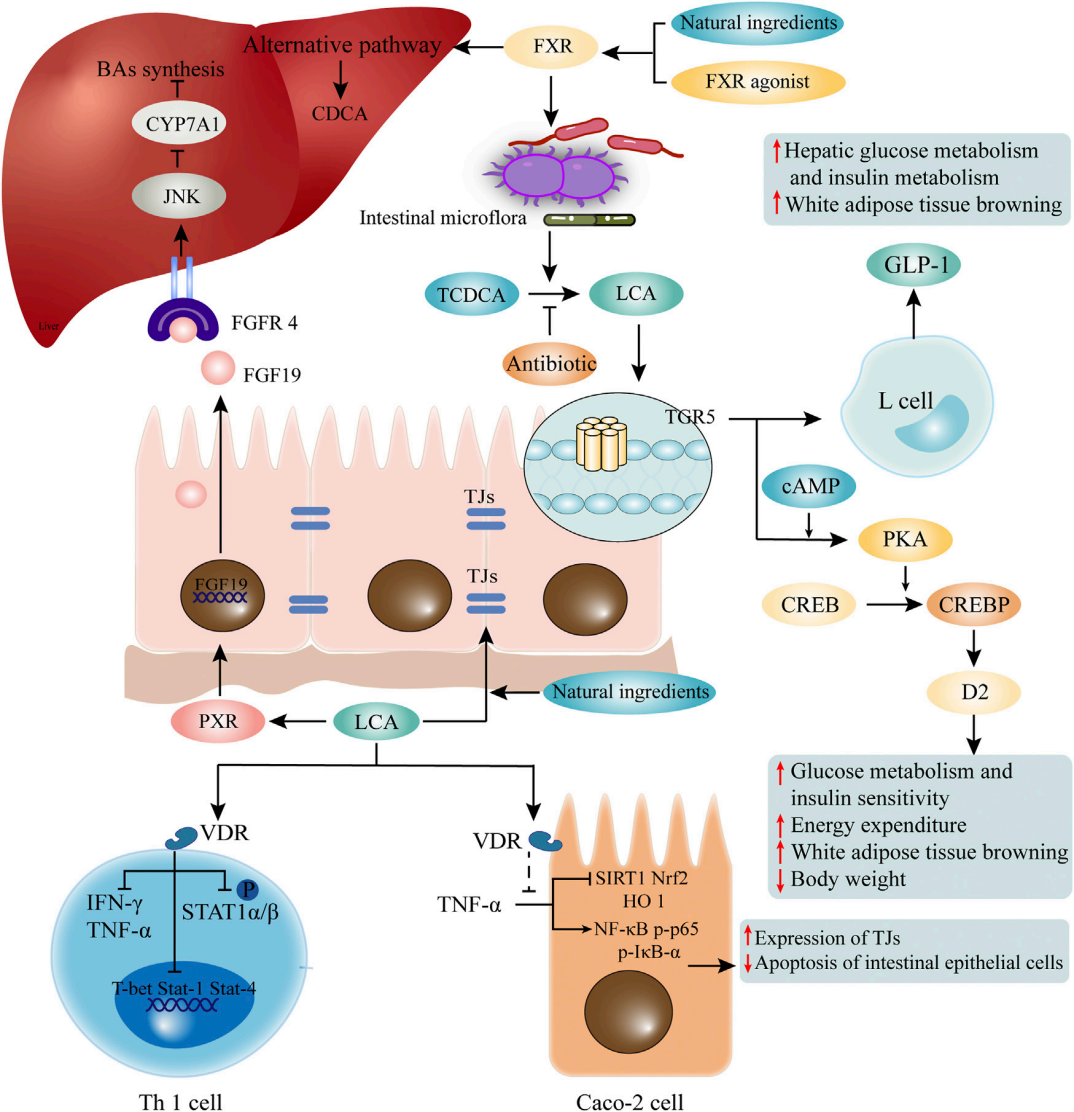
LCA mediated receptors and pathways. LCA mediates its functions through corresponding receptors. LCA activates VDR, and is involved in maintaining the function of TJs of intestinal epithelial cells and inhibition of Th1 cells. Natural ingredients can enhance VDR activation through LCA. Elevated LCA can activate TGR5, which promotes the secretion of GLP1 by intestinal epithelial cells, and participates in glucose metabolism and fat metabolism. The binding of LCA and PXR activates the transcription of FGF19. After recognition of FGFR4 on hepatocytes, it initiates the c-Jun N-terminal kinase (JNK) signaling pathway, which inhibits CYP7A1 and BA synthesis. In addition, activation of FXR induced by FXR agonist or some natural ingredients may change the composition of intestinal flora and promote the production of LCA. LCA, lithocholic acid; VDR, vitamin D receptor; TJs, tight junctions; TGR5, G protein-coupled receptor five; GLP1, glucagon-like peptide-1; PXR, pregnane X receptor; FGF19, fibroblast growth factor 19; FGFR4, FGF receptor four; CYP7A1, cholesterol 7α-hydroxylase.

The combination of LCA and VDR not only mediates its own detoxification, but also shows biological activities such as anti-inflammation and immune regulation. In Caco-2 cells, LCA inhibits TNF-α-mediated downregulation of silent information regulator 1 (SIRT1), nuclear factor erythroid 2-related factor 2 (Nrf2), and heme oxygenase 1 (HO 1), as well as the increase of NF-κB p-p65 and p-IκB-α (Yao et al., 2019). In CRC cells, LCA activates VDR to block NF-κB inflammatory signaling, and significantly reduces IL-1β -induced IL-8 secretion (Sun et al., 2008). LCA also regulates adaptive immunity and inhibits Th1 cell activation through VDR signaling (Pols et al., 2017). Although the concentration of free-form LCA in the peripheral circulation is only 0.5 μM (Steiner et al., 2011), it is sufficient to suppress the inflammatory response to Th1 cells (Pols et al., 2017). The free-form of LCA impede the activation of Th1 cells mainly in three ways: 1) inhibits the production of Th1 cytokines IFN-γ and TNF-α; 2) inhibits the expression of Th1 regulatory genes such as T-box protein (T-bet), Stat-1 and Stat-4 expressed in T cells; and 3) inhibits the phosphorylation of STAT1α/β (Pols et al., 2017) (Figure 2). Although the exact mechanisms are unknown, it recently has been found that LCA at physiological concentration inhibits ERK-1/2 phosphorylation and increases the basal phosphorylation level of p38 in Jurkat T cells (Pols et al., 2017). And phosphorylation of p38 signaling in T cells has been reported to induce T cell apoptosis, thereby suppressing chronic inflammation (Lanna et al., 2014). Unfortunately, the exact mechanisms by which LCA inhibits ERK phosphorylation and promotes P38 phosphorylation are still unknown.

### LCA and TGR5

As a member of the G protein-coupled receptor family, TGR5 is a cell membrane receptor of LCA. TGR5 expression is widely distributed in endocrine glands, adipocytes, muscles, immune organs, gallbladder and enteric nervous system (Marki, 1966). It has been reported that LCA and its analogue TLCA are the most potent endogenous ligands for TGR5 due to their hydrophobicity (Kawamata et al., 2003; Nguyen and Bouscarel, 2008; Sato et al., 2008). The EC_50_ value of LCA on TRG5 is reported to be 0.58 μM (Sato et al., 2008). Activation of TGR5 leads to receptor internalization, increased levels of cyclic adenosine monophosphate (cAMP), protein kinase A (PKA) activation, and increased levels of phosphorylation of target proteins (Li et al., 2018). The LCA induced TGR5 activation is broad and cell-specific, including the anti-inflammatory effects on macrophages, increased energy expenditure in brown adipose tissue (BAT), improved glucose metabolism and insulin sensitivity, gallbladder relaxation, and promotion of intestinal motility (Watanabe et al., 2006; Thomas et al., 2009).

In C57Bl/6J mice, LCA treatment significantly reduces body weight (Ward et al., 2017), which might be related to the effect of LCA on energy expenditure and fat metabolism. LCA binds to TGR5 on intestinal epithelial cells to activate the cAMP-D2 signaling pathway and promote energy expenditure (Watanabe et al., 2006). Enhancing the expression of TGR5 downstream thermogenic genes in BAT reduces the uptake of free fatty acid (FFA) in the liver (Fan et al., 2022). More surprisingly, the most potent TGR5 agonist developed by Yu *et al.*, 23(S)-methyl-LCA (23(S)-m-LCA), was three times more active than LCA at a concentration of 5 μM, but glucagon-like peptide-1 (GLP-1) transcripts in the mouse intestine increased nearly 26-fold (Yu et al., 2015). These results suggest that LCA is involved in mediating energy expenditure and GLP-1 secretion via TGR5 signaling.

Some natural components are also involved in the regulation of glucose metabolism by LCA and TGR5. As previously mentioned, natural ingredients can alter the structure of the gut microbiota and promote LCA production. Resveratrol can increase the LCA level, which activates TGR5 and up-regulate uncoupling protein one expression, thus accelerating energy expenditure (Chevalier et al., 2015; Hui et al., 2020b). Curcumin and capsaicin also rely on the reshaping of intestinal flora to promote LCA production, and the subsequent activation of TGR5 mediated cAMP/PKA signaling in maintaining glucose homeostasis (Hui et al., 2020a; Han et al., 2021). It is important to note that the beneficial effects of these natural ingredients on glucose intolerance are partially eliminated by the application of antibiotics, probably due to the inhibition of LCA-producing bact**e**ria (Figure 2).

An *in vitro* assay has demonstrated that LCA can inhibit the production of PiC in macrophages through activation of TGR5 (Yoneno et al., 2013). As mentioned previously, the anti-inflammatory effects of LCA on intestinal inflammation in ulcerative colitis patients are also partially dependent on the TGR5 signaling (Sinha et al., 2020). More importantly, the anti-cancer effect of LCA has been linked to TGR5 in studies of cancer cells from different tissues (Arnould et al., 2002; Astanehe et al., 2008; Arlia-Ciommo et al., 2014).

### LCA and FXR, PXR

Dysregulation of lipid metabolism has been linked to cancer, as cancer cells often exhibit a larger lipid requirement to meet their uncontrolled proliferation and metastasis (Luu et al., 2018). Moreover, the proliferation and metastasis of cancer cells are associated with increased exogenous lipid uptake, as well as the endogenous synthesis of lipids (Beloribi-Djefaflia et al., 2016). FXR activation is important in the regulation of lipid metabolism and glucose metabolism (Ding et al., 2015). Unlike TGR5, FXR inhibits GLP-1 transcriptional activity by promoting miR-33 expression and repressing its downstream targets glucagon (GCG) and CREBP1 (Li et al., 2019). While the LCA derivative 7-ethylidene-lithocholic acid effectively inhibits FXR-induced gene expression in hepatocytes and activates TGR5 to stimulate GLP-1 secretion from enteroendocrine cells, synergistical effects are achieved in maintaining glucose homeostasis (Stefela et al., 2021).

Activation of intestinal FXR by fexaramine (an intestine-restricted FXR agonist) can shape the intestinal microbiota to induce the conversion of TCDCA to LCA by *Acetatifactor* and *Bacteroides*, and the elevation of LCA then activates intestinal TGR5, which then stimulates intestinal “L" cells to secrete GLP-1 (Trabelsi et al., 2015), thereby modulates glucose metabolism and white adipose tissue browning. However, antibiotic treatment reverses the beneficial metabolic effects of fexaramine in obese and diabetic mice (Pathak et al., 2018). In addition, as mentioned above, natural ingredients can also promote LCA production by activating FXR or altering the gut microbiome. However, different from current FXR agonists, natural ingredients (e.g. grape seed proanthocyanidin) activate the gut FXR signaling and increase gene expression of BA synthetases of alternative pathway, contributing to the increased LCA production rather than modulation of the gut microbiome (Wu et al., 2021) (Figure 2). It has also been reported that activation of intestinal FXR signaling by LCA facilitates the reduction of inflammatory cytokines such as TNF-α, IL-1β and IL-6 in the ileum and serum, and protects the intestine against inflammation (Wu et al., 2021).

PXR has been shown to play an important role in the induction of phase I-III genes that are involved in the metabolism, transport and excretion of a variety of metabolites (Xing et al., 2020). PXR can only be activated by LCA and its derivative 3-keto-LCA, but does not respond to CDCA, DCA, or CA. The binding of LCA and PXR induces activation of the fibroblast growth factor 19 (FGF19) promoter (Wistuba et al., 2007). Intestinally secreted FGF19 binds to FGF receptor 4 (FGFR4) on hepatocytes and initiates the c-jun N-terminal kinase (JNK) signaling, which inhibits CYP7A1 and BA synthesis (Wistuba et al., 2007). Recent studies have reported that intestinal PXR regulates the expression of NF-κB and may be protect the mice from IBD (Okamura et al., 2020), but it remains to be determined whether LCA or 3-keto-LCA is involved in activation of hepatic and intestinal PXR.

The processing of Chinese medicinal herbs is believed to promote the absorption of the active ingredients (Chen et al., 2018). A recent study found that frankincense made with vinegar increases LCA level, activates constitutional androstane receptor and PXR, and upregulates the expression of drug absorption-associated protein in the liver, such as multidrug resistance-associated protein 2 (MRP2) and organic anion transporting polypeptide 1B3 (OATP1B3). Meanwhile, LCA activates PXR expression in the colon, which also promotes the expression of MRP2 and OATP1B3, thus facilitating drug absorption (Peng et al., 2022).

## Conclusion and Perspectives

In summary, LCA shows considerable protective effects on the intestinal environment, including maintaining the stability of TJs, and anti-bacterial and anti-inflammatory effects. Moreover, LCA can up-regulate the expression of absorption-related proteins, promote the absorption of herbs, and mediate the biological functions of these herbs in regulating glucose metabolism, lipid metabolism, and energy homeostasis. The utilization of the beneficial effects of LCA is an attractive topic.

Focusing on the molecular mechanisms of the anti-apoptotic and anti-inflammatory effects of LCA, the proper dosage as well as the intestinal environment should be taken into consideration for drug development. In addition, the significant differences in the composition of intestinal microorganisms and BA composition between humans and mice may also affect the validity of the conclusions obtained so far in the relevant studies. Gut microbiota is crucial for LCA transformation, while diet and some natural products can also affect the transformation. Therefore, a healthy microbial ecology is critical for maintaining BA homeostasis. The composition of the microbiota, changes in BA composition, and the interaction between LCA and related nuclear receptors and TGR5 signaling appear to be highly promising for the treatment of intestinal and hepatic diseases, as well as certain metabolic-related disorders.
